# Role of Hyaluronic Acid and Chondroitin Sulphate in Protecting Urinary Epithelium in Patients Operated for Differentiated Thyroid Cancer Undergoing Ablation Therapy with Iodine-131

**DOI:** 10.3390/cancers17193154

**Published:** 2025-09-28

**Authors:** Giuseppe Campagna, Chiara Lauri, Tiziana Lanzolla, Anna Festa, Luciano Carideo, Alberto Signore

**Affiliations:** 1 Nuclear Medicine Unit, Department Medical-Surgical Sciences and of Translational Medicine, Faculty of Medicine and Psychology, “Sapienza” University of Rome, 00189 Rome, Italy; chiara.lauri@uniroma1.it (C.L.); alberto.signore@uniroma1.it (A.S.); 2 Nuclear Medicine Unit, University Hospital “Sant’Andrea”, 00189 Rome, Italy; tlanzolla@ospedalesantandrea.it (T.L.); anna.festa@osepdalesantandrea.it (A.F.)

**Keywords:** ablation therapy, bacteriuria, cystitis, differentiated thyroid cancer, epithelial cells, iodine-131, urinary tract infection

## Abstract

**Simple Summary:**

The administration of hyaluronic acid and chondroitin sulphate has shown protective properties on the urinary epithelium in several different clinical conditions. We, therefore, explored the possible role of hyaluronic acid and chondroitin sulphate in preventing iodine-131-induced urinary infections in patients treated for thyroid cancer, while assessing whether urinary infections are related to doses of iodine-131 or patients’ preparation. To these purposes, several urine parameters were analyzed at basal time and up to one week. Our results show that urinary infections occur in a relevant percentage of patients undergoing iodine-131, either in patients who were treated with hyaluronic acid and chondroitin sulphate or in patients who were not. Nevertheless, in patients who experienced urinary infections, the use of hyaluronic acid and chondroitin sulphate was able to reduce the epithelial cells and bacteria count in urine. Therefore, similarly to other different clinical conditions, the use of hyaluronic acid and chondroitin sulphate could be a valid option in patients undergoing iodine-131 for thyroid cancer ablation to mitigate urinary infections.

**Abstract:**

Background: It is not clear if the administration of iodine-131 in patients with differentiated thyroid cancers induces relevant cystitis and lower urinary tract infections (UTIs). The aim of this study was to evaluate the impact of oral administration of hyaluronic acid and chondroitin sulphate (HA&CS) on the frequency of UTIs induced by iodine-131. Methods: In this study, 166 female patients with a surgical diagnosis of differentiated thyroid cancer who were hospitalized in Sant ’Andrea Hospital of Rome to undergo the first cycle of therapy with iodine-131 for thyroid remnant ablation were evaluated. Sixty-one patients were treated with HA&CS for 8 days, and thirty-nine patients were treated with HA&CS for 17 days. Sixty-six patients were not treated with HA&CS and were used as control subjects. Patients underwent urinalysis at different time points: immediately before ^131^I therapy, two days later, and one week later. Patients were divided in six groups based on whether they received the treatment with HA&CS, its duration, and whether they developed a UTI: group A, *n* = 51 patients who did not receive HA&CS and did not develop UTI; group B1, *n* = 37 patients treated with HA&CS for 8 days and did not develop UTI; group B2, *n* = 29 patients treated with HA&CS for 17 days and did not develop UTI; group C, *n* = 15 patients who did not receive HA&CS and developed UTI; group D1, *n* = 24 patients treated with HA&CS for 8 days and developed UTI; group D2, *n* = 10 patients treated with HA&CS for 17 days and developed UTI. Results: The bacteria count, red blood cells and white blood cells did not change when group A was compared to both group B1 and group B2, while pH and epithelial cells showed a decrease over time when comparing group A and group B2 (*p* = 0.01 and *p* < 0.0001, respectively). Comparing group C with group D1 and group D2, patients who did not receive HA&CS (group C) showed a higher bacteria and epithelial cell count compared to those who were pre-treated with HA&CS. Conclusions: HA&CS could be an effective supportive therapy to prevent and mitigate UTIs in patients treated with ^131^I.

## 1. Introduction

Thyroid cancer is by far the most common endocrine tumour, and its incidence is constantly rising [1]. The most frequent histotypes are well-differentiated thyroid cancers (DTCs), which include papillary thyroid cancer and follicular thyroid cancer, that account for about 90% of malignant thyroid tumours [2]. DTC benefits from nuclear-medicine therapy with ^131^I, either using low doses, with the purpose to ablate residual thyroid tissue after total or near-total thyroidectomy, or high doses aiming to treat any possible locoregional recurrences or lymph nodal, bone or pulmonary iodine-avid metastases [3,4]. When orally administered, iodine-131 is eliminated from the human body mainly through the urinary tract (37–75%), partly from fecal elimination (10%) and triflingly through sweat.

Therefore, not surprisingly, the ureters, the bladder and the urethra are the tissues receiving the highest radiation dose, followed by the kidneys [5,6,7]. Despite this, urinary side effects are rarely reported, with the most relevant adverse effects of radioiodine therapy being nausea, neck pain, salivary and lacrimal gland dysfunction and altered taste. Subacute and late adverse effects can be infertility, sialoadenitis, nasolacrimal duct obstruction and second primary malignancy [6,7,8,9], mostly depending on cumulative radiation dose.

Although rarely reported by patients, we hypothesized that sub-clinical or clinically relevant cystitis and lower urinary tract infections (UTIs) may represent a possible side effect of radioiodine therapy, particularly in female patients.

Indeed, nothing is known about the frequency and severity of UTIs in patients treated with ^131^I, nor if there is a dose-dependent effect, nor if the use of recombinant human thyroid-stimulating hormone (rhTSH) may increase or decrease such frequency, since it accelerates urinary elimination of free ^131^I. Furthermore, taking into account the prevalence of DTC and that many patients undergo radioiodine therapy after surgery, and considering that UTIs often may remain unknown and thus not treated, it could be clinically relevant to investigate how to prevent these side effects.

Radiation cystitis symptoms include a persistent urge to void, burning sensation during voiding, pollakiuria, pelvic discomfort, feeling pressure in the lower abdomen, hematuria and urine smell alterations.

The administration of hyaluronic acid and chondroitin sulphate (HA&CS) has shown protective properties on urinary epithelium in several different clinical conditions [10,11,12,13,14,15,16,17,18]. Amongst this class, Ialuril^®^ Soft Gels (IBSA, Lodi (LO) Italy) is a gluten-free and lactose-free dietary supplement, based on curcumin, quercetin, hyaluronic acid and chondroitine sulphate, which shows anti-inflammatory and analgesic properties and has the ability to create a protective biofilm on the urothelium, thus preventing bacteria attachment to uroepithelium [12]. In particular, the presence of curcumin has antioxidant properties, while quercetin, a flavonol abundant in fruit and vegetables, allows the intestinal absorption of curcumin and also exerts inflammatory and antibacterial effects [19,20,21,22].

The improvement of urinary symptoms and acute genito-urinary toxicity by the administration of HA&CS has been confirmed by several studies [10,11,23,24,25,26,27].

Since the oxidative stress with REDOX homeostasis imbalance has been largely described after ^131^I treatment for DTC [28,29,30], attention is focused on the use of antioxidants to prevent ^131^I side effects [31,32].

The aims of our study were as follows: to examine the frequency of UTIs in patients with DTC undergoing ^131^I therapy; to assess if UTIs are ^131^I dose-dependent; to determine if the preparation of patients for ^131^I therapy with hypothyroidism or rhTSH may influence the frequency of UTIs and, finally, to explore if the administration of HA&CS can reduce the frequency of UTIs or urinary tract epithelium alterations induced by ^131^I.

## 2. Patients and Methods

### 2.1. Study Design and Patients

We retrospectively evaluated a total of 166 female patients (from 20 to 80 years, mean age: 49.8 years) with surgical diagnosis of DTC (both papillary and follicular histotypes) who were hospitalized in the Nuclear Medicine Unit of Sant’Andrea Hospital of Rome between October 2015 and November 2019 to undergo the first cycle of therapy with iodine-131 for remnant ablation purposes. Male patients were excluded because they are usually less subject to lower UTIs than females for anatomic/physiologic reasons, as also shown by a pilot analysis that we previously performed. We also excluded all those patients with a history of congenital or surgical urinary tract alterations, patients with renal failure, urinary tract obstruction or with a neurogenic bladder.

Patients received both low (50–80 mCi; *n* = 80 patients) and high-doses (100, 120 and 150 mCi; *n* = 86 patients) of ^131^I based on post-surgical staging. According to guidelines [2,5], for patient preparation to radioiodine administration, 120 subjects received rhTSH injections for two days before therapy, while the other 46 patients underwent thyroid hormone withdrawal.

**Figure 1:**
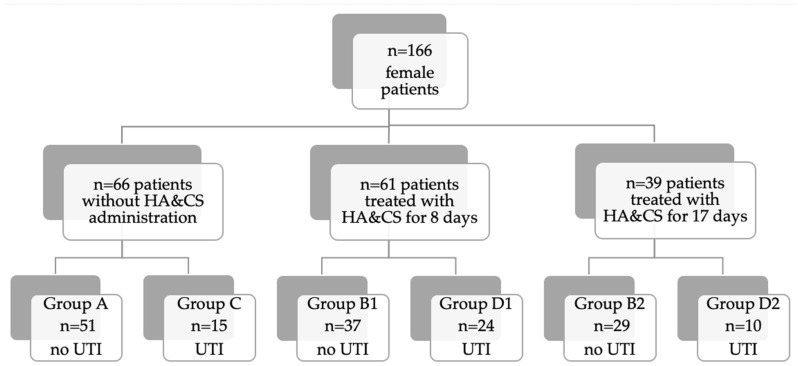
Flow-chart of the population.

We divided our population in six groups (Figure 1): group A (*n* = 51) patients who underwent radioiodine therapy without HA&CS administration and did not develop UTI; group B1 (*n* = 37) patients treated with two capsules of HA&CS per day (morning and evening) starting the day before radioiodine therapy and for the following 7 days (8 days total) and did not develop UTI; group B2 (*n* = 29) patients who received two capsules of HA&CS per day starting 10 days before therapy until one week after (17 days total) and did not develop UTI; group C (*n* = 15) patients who underwent radioiodine therapy without HA&CS and developed UTI; group D1 (*n* = 24) patients who received two capsules of HA&CS for 8 days total and developed UTI and group D2 (*n* = 10) patients who received two capsules of HA&CS for 17 days total and developed UTI.

Moreover, we analyzed three further groups: groups A + C (*n* = 66), groups B1 + D1 (*n* = 61) and groups B2 + D2 (*n* = 39) to assess the differences in UTI development, according to the treatment with HA&CS and its duration.

Finally, we performed additional analyses to assess whether the infection is dependent on ^131^I dose or patients’ preparation (rhTSH vs. hypothyroidism).

### 2.2. Urine Analysis

Urine analysis was performed prior to therapy at the time of hospitalization, two days after radioiodine administration and one week after therapy. Bacteria count, epithelial cells (EPs), pH, red blood cells (RBCs), and white blood cells (WBCs) were collected at each time point.

### 2.3. Ethical Aspects

Our study included a retrospective analysis of data acquired and recorded in an internal archive of the Nuclear Medicine Unit of Sant’Andrea Hospital of Rome; the local Ethics Committee was notified on 10 February 2015. Since hyaluronic acid was already commercially available and used in clinical practice, a protocol number was not required. Each patient provided informed consent, dated and signed. Patients’ data were anonymized and included in a database.

### 2.4. Statistical Analysis

Continuous variables were shown as mean ± standard deviation (SD) and at a 95% confidence interval (95%CI).

Comparisons of urine parameters between hypothyroidism and rhTSH were evaluated by the Student *t* test (verified normality) and the Welch test (failed normality).

Longitudinal data analysis of bacteria, epithelial cells (EPs), pH, RBCs, and WBCs and the comparison between the groups was performed using a Generalized Linear Mixed Model method (GLIMMIX) with repeated measures, along with the distribution of data fixed as Gaussian.

The distribution data (urine parameters) and residuals were tested by the Shapiro–Wilk test and checking the Q-Q plot, while the homoscedasticity was verified by checking the studentized residuals vs. the fitted values plot.

A *p*-value < 0.05 was considered statistically detectable. All statistical analyses and the graphics were performed using SAS v.9.4 and/or JMP Pro v. 16.0 (SAS Institute Inc., 100 SAS Campus Drive, Cary, NC, 27513-2414, USA).

## 3. Results

Overall, in our series, a lower UTI was observed in 49 out of 166 patients (29.5%) and, in particular, in 15 out of 66 patients (22.7%) who were not treated with HA&CS and in 34 out of 100 patients who were treated (39.3% in the group treated for 8 days and 25.6% in the group treated for 17 days, respectively) without no significant differences neither between pre-treated and non-pretreated groups (*p* = 0.12), nor according to HA&CS duration (*p* = 0.16).

No differences in urine parameters were observed in non-infected patients regardless of the ^131^I dose (Table 1) or their preparation (with rhTSH or hypothyroidism (Table 2 and Table 3)).

When comparing non-infected patients without HA&CS pre-treatment (group A; mean age ± SD: 53.06 ± 12.97; min: 19.41 and max: 75.54 years) and non-infected patients with HA&CS for 8 days (group B1; mean age ± SD: 52.78 ± 13.84; min: 19.07 and max: 81.14 years), no detectable differences were observed in terms of pH, bacteria, EP, RBC and WBC count. When comparing group A and group B2 (non-infected patients with HA&CS for 17 days; mean age ± SD: 51.91 ± 13.18; min: 19.64 and max: 72.85 years), bacteria, RBC and WBC count were not statistically different, while EP and pH values were statistically higher in group A (*p* = 0.01, *p* < 0.0001, respectively).

When comparing infected patients without HA&CS pre-treatment (group C; mean age ± SD: 41.09 ± 9.39; min: 23.39 and max: 57.45 years), and infected patients with HA&CS for 8 days (group D1; mean age ± SD: 46.02 ± 13.04; min: 25.82 and max: 81.66 years), bacteria and EP count were statistically higher in non-treated patients (*p* < 0.0001 and *p* = 0.002, respectively) and bacteriuria was higher at both the second day (group C: 2.80 ± 0.79; 95%CI: 2.24 to 3.36 vs. group D1: 1.79 ± 0.78; 95%CI: 1.46 to 2.12, *p* = 0.002) and at the seventh day (group C: 2.90 ± 1.20; 95%CI: 2.04 to 3.76 vs. group D1: 1.50 ± 0.51; 95%CI: 1.28 to 1.72, *p* = 0.0009).

Group C also showed a statistically higher bacteria and EP count compared to infected patients with HA&CS for 17 days (group D2; mean age ± SD: 36.43 ± 10.37; min: 26.51 and max: 58.97 years) (*p* = 0.0005 and *p* = 0.001, respectively). Bacteria count was statistically higher at any time point: group C (baseline): 2.50 ± 0.53; 95%CI: 2.12 to 2.88 vs. group D2 (baseline): 1.70 ± 0.48; 95%CI: 1.35 to 2.05, *p* = 0.009; group C (second day): 2.80 ± 0.79; 95%CI: 2.24 to 3.36 vs. group D2 (second day): 1.50 ± 0.53; 95%CI: 1.12 to 1.88, *p* = 0.007; group C (seventh days): 2.90 ± 1.20; 95%CI: 2.04 to 3.76 vs. group D2 (second days): 1.80 ± 0.42; 95%CI: 1.50 to 2.10, *p* = 0.02.

By comparing non-treated patients with and without UTI (group A + C; mean age ± SD: 50.34 ± 13.19; min: 19.41 and max: 75.54 years) and patients treated with HA&CS for 8 days, both infected and non-infected (groups B1 + D1; mean age ± SD: 50.12 ± 13.83; min: 19.07 and max: 81.66 years), we did not observe any detectable difference in terms of bacteria, EP, pH, RBC and WBC count.

By comparing non-treated patients with and without UTI (groups A + C) and patients treated with HA&CS for 17 days, both infected and non-infected (groups B2 + D2; mean age ± SD: 47.94 ± 14.16; min: 19.64 and max: 72.85 years), EP and pH count were higher in non-treated patients (*p* = 0.008 and *p* < 0.0001, respectively), while no significant differences were observed in terms of bacteria, RBC and WBC count. At seventh day non-HA&CS-treated patients showed statistically higher bacteriuria (1.54 ± 1.01; 95%CI: 1.28 to 1.80 vs. 1.21 ± 0.41; 95%CI: 1.07 to 1.34, *p* = 0.03).

**Figure 2:**
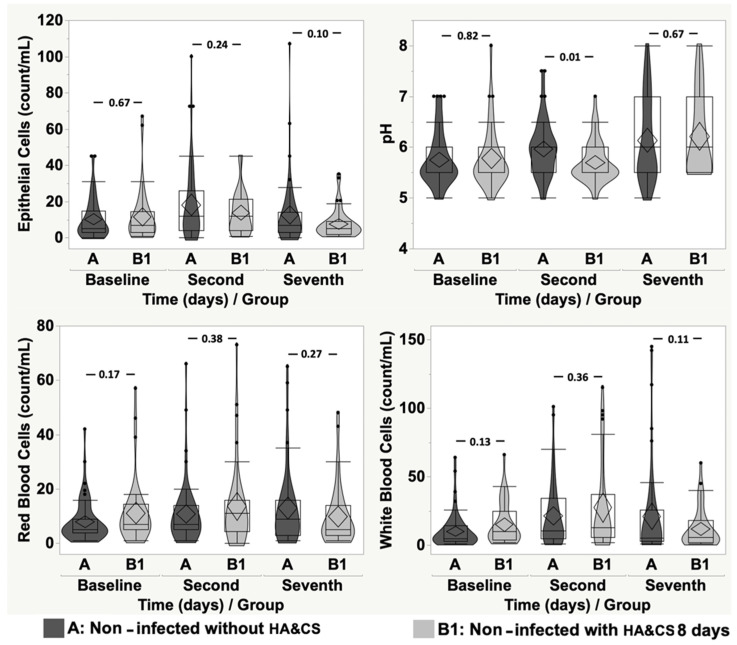
Violin-box-plot of epithelial cells, pH, red blood cells, and white blood cells according to time in “non-infected without HA&CS” vs. “non-infected with HA&CS for 8 days”.

**Figure 3:**
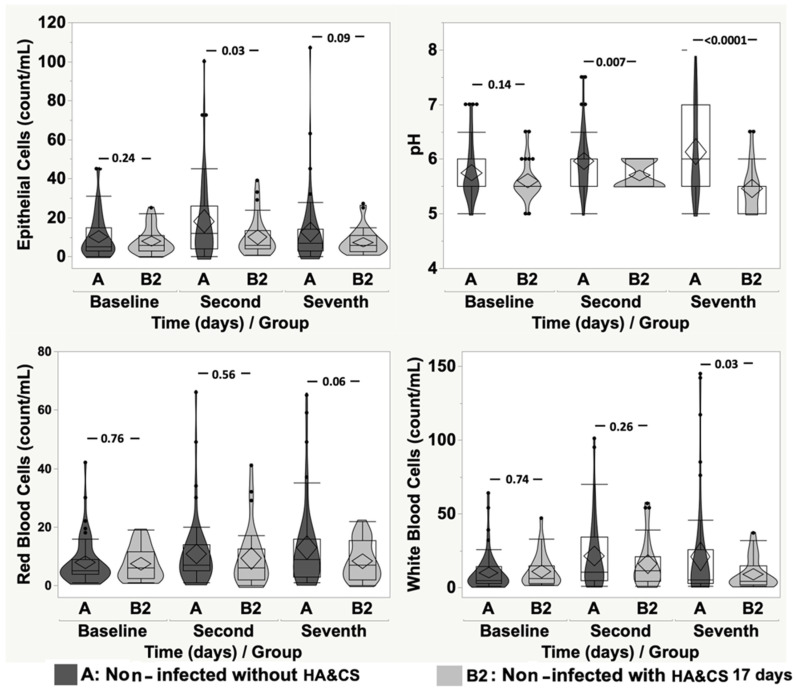
Violin-box-plot of epithelial cells, pH, red blood cells and white blood cells according to time in “non-infected without HA&CS” and “non-infected with HA&CS for 17 days”.

Figure 2 and Figure 3 show the comparisons between group A and group B1 and B2, respectively, at baseline, second day, and seventh day, in terms of EP, pH, RBC and WBC count. Between group A and group B1, only pH showed a detectable difference at the second day (*p* = 0.01), while in all other temporal comparisons, we did not observe statistically relevant differences in terms of EP, pH, RBC, and WBC count (Figure 2). When comparing group A and B2 (Figure 3), relevant differences were detected for EP (*p* = 0.03) and pH (*p* = 0.007) at the second day, and for pH (*p* < 0.0001) and WBC (*p* = 0.03), at the seventh day.

**Figure 4:**
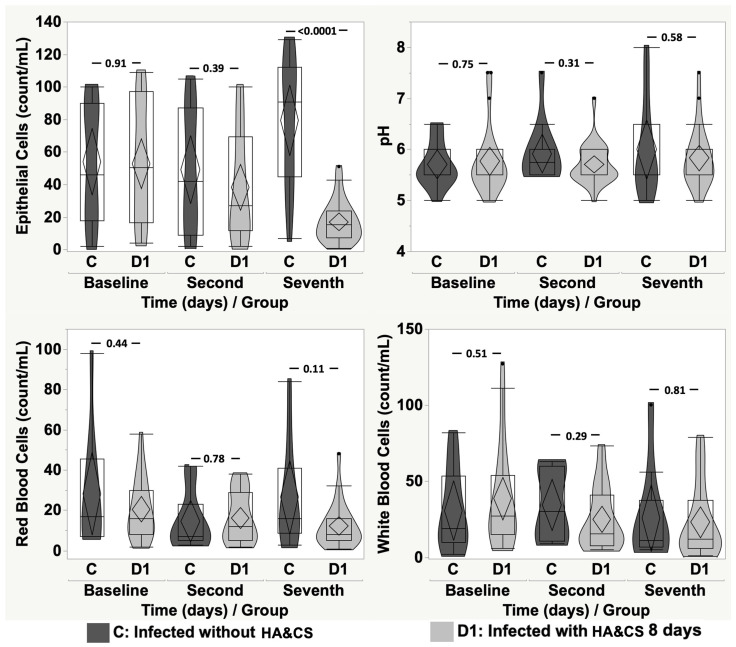
Violin-box-plot of epithelial cells, pH, red blood cells and white blood cells according to time in “infected without HA&CS” vs. “infected with HA&CS for 8 days”.

**Figure 5:**
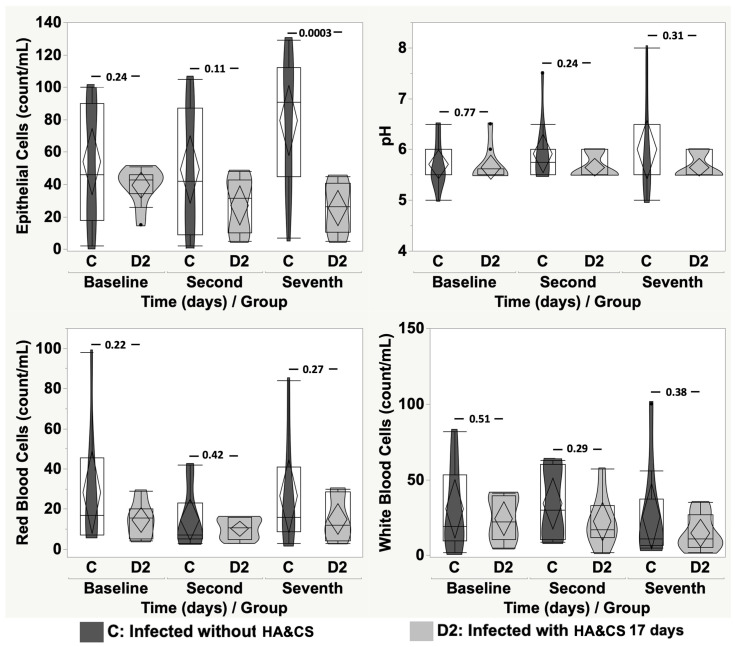
Violin-box-plot of epithelial cells, pH, red blood cells and white blood cells according to time in “infected without HA&CS” vs. “infected with HA&CS for 17 days”.

Figure 4 and Figure 5 show the comparison between group C and group D1 and D2, respectively, at baseline, on the second day and seventh day, in terms of EP, pH, RBC and WBC. Only EP showed a detectable difference on the seventh day when comparing group C with group D1 and D2 (*p* < 0.0001 and *p* = 0.0003, respectively).

**Figure 6:**
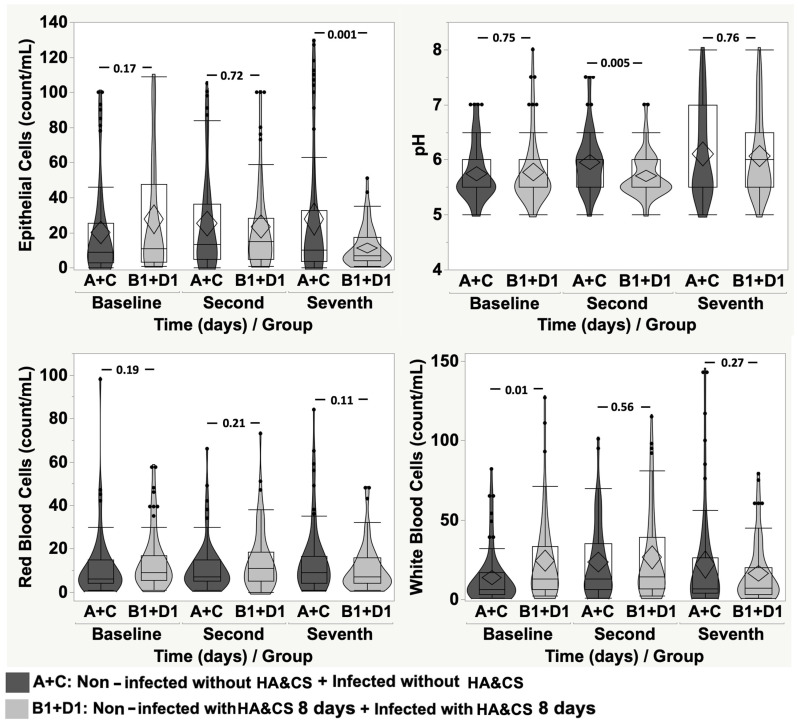
Violin-box-plot of epithelial cells, pH, red blood cells, and white blood cells according to time in “non-infected without HA&CS + infected without HA&CS” vs. “non-infected with HA&CS for 8 days + infected with HA&CS for 8 days”.

**Figure 7:**
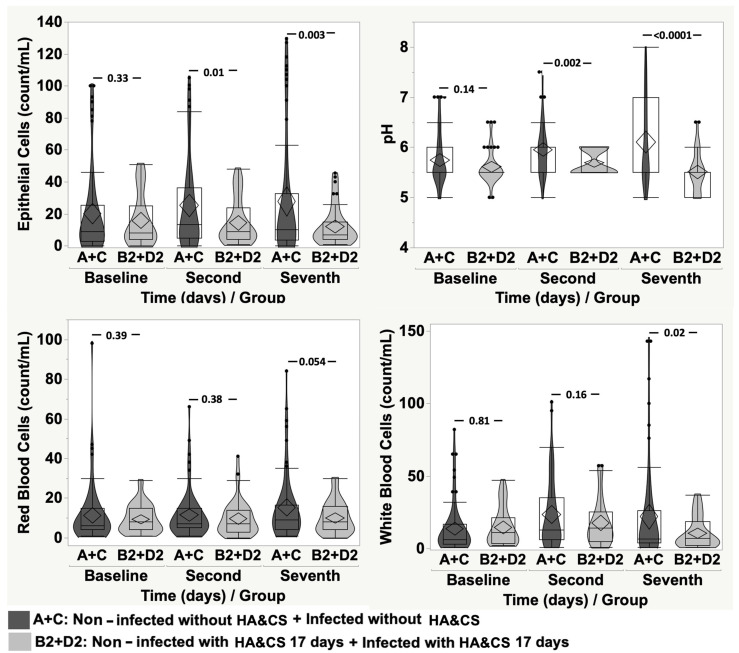
Violin-box-plot of epithelial cells, pH, red blood cells and white blood cells according to time in “non-infected without HA&CS + infected without HA&CS” vs. “non-infected with HA&CS for 17 days + infected with HA&CS for 17 days”.

Comparing groups A + C and groups B1 + D1, we observed a difference for EP (*p* = 0.001) on the seventh day, for pH (*p* = 0.005) on the second day and at baseline for WBC (*p* = 0.01) (Figure 6). Comparing groups A + C and groups B2 + D2, again, we observed differences for EP and pH on the second day (*p* = 0.01 and *p* = 0.002, respectively) and on the seventh day (*p* = 0.003 and *p* < 0.0001, respectively) and for WBC on the seventh day (*p* = 0.02) (Figure 7).

All the groups were matched by age; indeed, comparing the age for each group, no statistically detectable differences were observed (A vs. B1, *p* = 0.92; A vs. B2, *p* = 0.71; C vs. D1, *p* = 0.21; C vs. D2, *p* = 0.26; A + C vs. B1 + D1, *p* = 0.93; A + C vs. B2 + D2, *p* = 0.38).

## 4. Discussion

With the increase in life expectancy and of diagnostic procedures, the number of thyroidectomies and ^131^I treatments has rapidly grown, and the attention has shifted to preventing or reducing ^131^I side effects. It is now more clear that DTC patients need a multidisciplinary approach, especially in patients with several comorbidities, and this should also include the adoption of anti-oxidative compounds or diet to reduce REDOX imbalance induced by ^131^I [28,29,33].

To the best of our knowledge, this is the first study addressing the possible role of HA&CS in reducing the risk of UTI in patients undergoing both high and low doses of ^131^I for thyroid remnant ablation.

Despite a relevant proportion of patients treated with HA&CS who developed a UTI, our results show that HA&CS is able to reduce EP and bacteria count both within 8 days and within 17 days of administration in infected patients.

Also, the pH is affected by the positive effect of the use of HA&CS; indeed, when considering the non-infected patients, those who received HA&CS for 17 days showed lower pH values; when comparing the combination of both infected and non-infected patients without HA&CS (groups A + C) and both infected and non-infected patients with HA&CS for 17 days (groups B2 + D2), the administration of HA&CS was associated with lower pH values.

This study has several limitations: first of all, it is a retrospective single-centre study; therefore, external validity and generalizability might be hampered. Moreover, potential confounders, such as possible baseline differences regarding the risk factors for UTI (diabetes, hydration status, prior catheter use, etc.), were not considered, thus possibly introducing a detection bias. In addition to this, urinalysis up to one week post-^131^I therapy might not be sufficient to detect possible longer-term effects on UTI incidence in patients undergoing radio-metabolic therapy for thyroid remnant ablation. Finally, we did not systematically explore urinary discomfort or pain in patients who developed UTI, thus we cannot reach any conclusion on the possible role of HA&CS on patients’ quality of life.

Nevertheless, despite these limitations, our results show that the effect of HA&CS on EP and bacteria counts is evident after 8 days and is maintained at 17 days. Based on these results, we could conclude that 8 days are enough to improve urinary parameters in UTI in patients operated on for differentiated thyroid cancer undergoing ablation therapy with ^131^I.

## 5. Conclusions

The use of HA&CS for 8 days in patients operated on for DTC undergoing ablation therapy with ^131^I leads to a reduction in EP and bacteria count in the urine of patients with lower UTI and could therefore be taken into consideration to mitigate urine parameters in patients with infection.

## Figures and Tables

**Table 1:** Temporal assessment of urine parameters in non-infected patients without HA&CS and treated with low dose of ^131^I versus non-infected patients without HA&CS and treated with high dose of ^131^I.

Parameter	A1 Non-Infected Without HA&CS Low Dose ^131^I			A2 Non-Infected Without HA&CS High Dose ^131^I			*p* (A1 vs. A2)
	Baseline Mean ± SD (95%CI)	Second Day Mean ± SD (95%CI)	Seventh Day Mean ± SD (95%CI)	Baseline Mean ± SD (95%CI)	Second Day Mean ± SD (95%CI)	Seventh Day Mean ± SD (95%CI)	
Bacteria (count)	1.00 ± 0.00 (NA to NA)	1.27 ± 0.55 (1.03 to 1.52)	1.27 ± 0.63 (0.99 to 1.55)	1.00 ± 0.00 (NA to NA)	1.34 ± 0.81 (1.04 to 1.65)	1.28 ± 0.80 (0.97 to 1.58)	0.81
Epithelial Cells (count/mL)	10.27 ± 9.02 (6.27 to 13.38)	20.23 ± 21.59 (10.65 to 29.80)	11.18 ± 15.56 (4.28 to 18.08)	10.38 ± 12.21 (5.73 to 15.02)	16.83 ± 20.47 (9.04 to 24.62)	13.76 ± 19.93 (6.18 to 21.34)	0.95
pH	5.80 ± 0.50 (5.57 to 6.02)	6.07 ± 0.58 (5.81 to 6.33)	6.14 ± 0.85 (5.76 to 6.51)	5.72 ± 0.56 (5.51 to 5.94)	5.90 ± 0.52 (5.70 to 6.10)	6.14 ± 0.85 (5.81 to 6.46)	0.57
Red Blood Cells (count/mL)	9.68 ± 9.51 (5.47 to 13.9)	12.45 ± 16.15 (5.30 to 19.61)	15.09 ± 18.28 (6.99 to 23.19)	6.79 ± 6.2 (4.43 to 9.15)	9.66 ± 7.21 (6.91 to 12.40)	11.55 ± 10.41 (7.59 to 15.51)	0.21
White Blood Cells (count/mL)	12.86 ± 17.88 (4.94 to 20.79)	16.86 ± 18.46 (8.68 to 25.05)	20.36 ± 37.29 (3.83 to 36.90)	8.29 ± 6.85 (5.63 to 10.94)	25.18 ± 26.71 (14.82 to 35.54)	21.93 ± 32.47 (9.34 to 34.52)	0.71

Bacteria: Baseline vs. Baseline, *p* = 0.99; Second Day vs. Second Day, *p* = 0.72; Seventh Day vs. Seventh Day, *p* = 0.99. Epithelial Cells: Baseline vs. Baseline, *p* = 0.97; Second Day vs. Second Day, *p* = 0.61; Seventh Day vs. Seventh Day, *p* = 0.95. pH: Baseline vs. Baseline, *p* = 0.66; Second Day vs. Second Day, *p* = 0.31; Seventh Day vs. Seventh Day, *p* = 0.99. Red Blood Cells: Baseline vs. Baseline, *p* = 0.25; Second Day vs. Second Day, *p* = 0.48; Seventh Day vs. Seventh Day, *p* = 0.45. White Blood Cells: Baseline vs. Baseline, *p* = 0.29; Second Day vs. Second Day, *p* = 0.23; Seventh Day vs. Seventh Day, *p* = 0.88.

**Table 2:** Assessment of urine parameters in non-infected patients without HA&CS pre-treatment.

Parameter	Hypothyroidism (*n* = 16) Mean ± SD, (95%CI)	rhTSH (*n* = 35) Mean ± SD, (95%CI)	*p*
Bacteria (count)	1.00 ± 0.00, (NE to NE)	1.00 ± 0.00, (NE to NE)	--
Epithelial cells (count/mL)	11.50 ± 7.77, (7.36 to 15.64)	9.80 ± 12.06, (5.66 to 13.94)	0.61
pH	5.87 ± 0.53, (5.59 to 6.16)	5.70 ± 0.53, (5.52 to 5.88)	0.28
Red Blood Cells (count/mL)	10.37 ± 11.52, (4.23 to 16.51)	6.97 ± 5.32, (5.14 to 8.80)	0.27
White Blood Cells (count/mL)	8.56 ± 9.89, (3.29 to 13.83)	11.12 ± 14.25, (6.14 to 16.09)	0.52

**Table 3:** Assessment of urine parameters in infected patients without HA&CS pre-treatment.

Parameter	Hypothyroidism (*n* = 5) Mean ± SD, (95%CI)	rhTSH (*n* = 10) Mean ± SD, (95%CI)	*p*
Bacteria (count)	2.40 ± 0.55, (1.72 to 3.08)	2.50 ± 0.53, (2.12 to 2.88)	0.74
Epithelial cells (count/mL)	85.40 ± 6.19, (77.72 to 93.08)	62.50 ± 31.97, (39.63 to 85.37)	0.053
pH	5.70 ± 0.27, (5.36 to 6.04)	5.70 ± 0.48, (5.35 to 6.04)	1.00
Red Blood Cells (count/mL)	30.60 ± 7.02, (21.88 to 39.32)	28.30 ± 28.92, (7.61 to 48.99)	0.82
White Blood Cells (count/mL)	30.40 ± 12.93, (14.34 to 46.46)	30.70 ± 27.21, (11.23 to 50.17)	0.98

## Data Availability

The original data presented in the study are available by writing to the first author.

## Abbreviations

^131^I	Iodine-131
95%CI	95% Confidence interval
DTC	Differentiated thyroid cancer
EPs	Epithelial cells
HA&CS	Hyaluronic acid and chondroitin sulphate
RBCs	Red blood cells
rhTSH	Recombinant human thyroid-stimulating hormone
UTIs	Urinary tract infections
WBCs	White blood cells

## References

[1] Matrone A., Campopiano M.C., Nervo A., Sapuppo G., Tavarelli M., De Leo S. (2020). Differentiated Thyroid Cancer, From Active Surveillance to Advanced Therapy: Toward a Personalized Medicine. Front. Endocrinol..

[2] Haugen B.R., Alexander E.K., Bible K.C., Doherty G.M., Mandel S.J., Nikiforov Y.E., Pacini F., Randolph G.W., Sawka A.M., Schlumberger M. (2016). 2015 American Thyroid Association Management Guidelines for Adult Patients with Thyroid Nodules and Differentiated Thyroid Cancer: The American Thyroid Association Guidelines Task Force on Thyroid Nodules and Differentiated Thyroid Cancer. Thyroid.

[3] Bal C.S., Padhy A.K. (2015). Radioiodine Remnant Ablation: A Critical Review. World J. Nucl. Med..

[4] Luster M., Clarke S.E., Dietlein M., Lassmann M., Lind P., Oyen W.J., Tennvall J., Bombardieri E. (2008). Guidelines for radioiodine therapy of differentiated thyroid cancer. Eur. J. Nucl. Med. Mol. Imaging.

[5] Andresen N.S., Buatti J.M., Tewfik H.H., Pagedar N.A., Anderson C.M., Watkins J.M. (2017). Radioiodine Ablation following Thyroidectomy for Differentiated Thyroid Cancer: Literature Review of Utility, Dose, and Toxicity. Eur. Thyroid. J..

[6] Hänscheid H., Lassmann M., Verburg F.A. (2023). Determinants of target absorbed dose in radionuclide therapy. Z. Für Med. Phys..

[7] O’Donoghue J., Zanzonico P., Humm J., Kesner A. (2022). Dosimetry in radiopharmaceutical therapy. J. Nucl. Med..

[8] Fard-Esfahani A., Emami-Ardekani A., Fallahi B., Fard-Esfahani P., Beiki D., Hassanzadeh-Rad A., Eftekhari M. (2014). Adverse effects of radioactive iodine-131 treatment for differentiated thyroid carcinoma. Nucl. Med. Commun..

[9] Van Nostrand D., Freitas J.E., Sawka A.M., Tsang R.W. (2016). Side effects of 131I for therapy of differentiated thyroid carcinoma. Thyroid Cancer: A Comprehensive Guide to Clinical Management.

[10] Fersino S., Fiorentino A., Giaj Levra N., Mazzola R., Ricchetti F., Di Paola G., Cavalleri S., Alongi F. (2016). Impact of Ialuril Soft Gels in reducing urinary toxicity during radical hypofractionated radiotherapy in prostate cancer: A preliminary experience. Minerva Urol. Nefrol..

[11] Manfredi C., Spirito L., Calace F.P., Balsamo R., Terribile M., Stizzo M., Romano L., Napolitano L., Califano G., Cirillo L. (2022). Oral Preparation of Hyaluronic Acid, Chondroitin Sulfate, Curcumin, and Quercetin (Ialuril® Soft Gels) for the Prevention of LUTS after Intravesical Chemotherapy. Pathophysiology.

[12] Riemma G., Vinci D., La Verde M., Caniglia F.M., Scalzone G., Torella M. (2025). Adding collagen, propolis plus quercetin, bacillus coagulans, hyaluronic acid and chondroitin sulphate to D-mannose avoids symptoms and prevents recurrence in women with recurrent urinary tract infections: A single-blind randomized controlled trial. Expert. Rev. Anti-Infect. Ther..

[13] Crocetto F., Balsamo R., Amicuzi U., De Luca L., Falcone A., Mirto B.F., Giampaglia G., Ferretti G., Capone F., Machiella F. (2023). Novel key ingredients in urinary tract health—The role of D-mannose, chondroitin sulphate, hyaluronic acid, and N-acetylcysteine in urinary tract infections (Uroial PLUS^®^). Nutrients.

[14] Stellavato A., Pirozzi A.V., Diana P., Reale S., Vassallo V., Fusco A., Donnarumma G., De Rosa M., Schiraldi C. (2019). Hyaluronic acid and chondroitin sulfate, alone or in combination, efficiently counteract induced bladder cell damage and inflammation. PLoS ONE.

[15] Torella M., Del Deo F., Grimaldi A., Iervolino S.A., Pezzella M., Tammaro C., Gallo P., Rappa C., De Franciscis P., Colacurci N.J. (2016). Efficacy of an orally administered combination of hyaluronic acid, chondroitin sulfate, curcumin and quercetin for the prevention of recurrent urinary tract infections in postmenopausal women. Eur. J. Obstet. Gynecol. Reprod. Biol..

[16] Boeri L., De Lorenzis E., Lucignani G., Turetti M., Silvani C., Zanetti S.P., Longo F., Albo G., Salonia A., Montanari E. (2024). Oral preparation of hyaluronic acid, chondroitin sulfate, N-acetylglucosamine, and vitamin C improves sexual and urinary symptoms in participants with recurrent urinary tract infections: A randomized crossover trial. J. Sex. Med..

[17] King G.K., Goodes L.M., Hartshorn C., Thavaseelan J., Jonescu S., Watts A., Rawlins M., Woodland P., Synnott E.L., Barrett T. (2023). Intravesical hyaluronic acid with chondroitin sulphate to prevent urinary tract infection after spinal cord injury. J. Spinal Cord. Med..

[18] Rahnama’i M.S., Javan Balegh Marand A., Röschmann-Doose K., Steffens L., Arendsen H.J. (2022). The efficacy and safety of intravesical chondroitin sulphate solution in recurrent urinary tract infections. BMC Urol..

[19] Shabbir U., Rubab M., Daliri E.B., Chelliah R., Javed A., Oh D.H. (2021). Curcumin, quercetin, catechins and metabolic diseases: The role of gut microbiota. Nutrients.

[20] Grill A.E., Koniar B., Panyam J. (2014). Co-delivery of natural metabolic inhibitors in a self-microemulsifying drug delivery system for improved oral bioavailability of curcumin. Drug Deliv. Transl. Res..

[21] Hamzah H., Hertiani T., Pratiwi S.U., Nuryastuti T. (2020). Efficacy of quercetin against polymicrobial biofilm on catheters. Res. J. Pharm. Technol..

[22] Güran M., Altundağ E.M., Şanlıtürk G., Ustürk S. (2025). In vitro Evaluation of the Combinatory Antibacterial and Antiproliferative Effects of Ciprofloxacin and Curcumin–Quercetin Mixture. Appl. Biochem. Microbiol..

[23] Gacci M., Saleh O., Giannessi C., Chini T., Della Camera P.A., Detti B., Livi L., Finazzi Agro E., Li Marzi V., Minervini A. (2016). Bladder Instillation Therapy With Hyaluronic Acid and Chondroitin Sulfate Improves Symptoms of Postradiation Cystitis: Prospective Pilot Study. Clin. Genitourin. Cancer.

[24] Nicosia L., Vitale C., Cuccia F., Figlia V., Giaj-Levra N., Mazzola R., Ricchetti F., Rigo M., Ruggieri R., Cavalleri S. (2022). Hypofractionated Postoperative Radiotherapy in Prostate Cancer with Ialuril Soft Gels^®^: Toxicity and Efficacy Analysis on a Retrospective Series of 305 Patients. Cancer Manag. Res..

[25] Salonia A. (2016). New preclinical data—The case for Ialuril^®^. Urologia.

[26] Morelli M., Mocciaro R., Venturella R., Albano A., Sacchinelli A., Zullo F. (2015). Hyaluronic acid-chondroitin sulfate: A potential factor to select pure stress urinary incontinence in patients with interstitial cystitis⁄painful bladder syndrome and mixed incontinence symptoms. Minerva Ginecol..

[27] Cicione A., Cantiello F., Ucciero G., Salonia A., Torella M., De Sio M., Autorino R., Carbone A., Romancik M., Tomaskin R. (2014). Intravesical treatment with highly-concentrated hyaluronic acid and chondroitin sulphate in patients with recurrent urinary tract infections: Results from a multicentre survey. Can. Urol. Assoc. J..

[28] Signore A., Campagna G., Marinaccio J., de Vitis M., Lauri C., Berardinelli F., Tofani A., Chianelli M., Borro M., Gentile G. (2022). Analysis of Short-Term and Stable DNA Damage in Patients with Differentiated Thyroid Cancer Treated with 131I in Hypothyroidism or with Recombinant Human Thyroid-Stimulating Hormone for Remnant Ablation. J. Nucl. Med. Off. Publ. Soc. Nucl. Med..

[29] Monteiro Gil O., Oliveira N.G., Rodrigues A.S., Laires A., Ferreira T.C., Limbert E., Léonard A., Gerber G., Rueff J. (2000). Cytogenetic alterations and oxidative stress in thyroid cancer patients after iodine-131 therapy. Mutagenesis.

[30] Buczyńska A., Sidorkiewicz I., Rogucki M., Siewko K., Adamska A., Kościuszko M., Maliszewska K., Kozłowska G., Szumowski P., Myśliwiec J. (2021). Oxidative stress and radioiodine treatment of differentiated thyroid cancer. Sci. Rep..

[31] Yang L., Ma J., Lei P., Yi J., Ma Y., Huang Z., Wang T., Ping H., Ruan D., Sun D. (2023). Advances in Antioxidant Applications for Combating 131I Side Effects in Thyroid Cancer Treatment. Toxics.

[32] Berti A.P., Düsman E., Mariucci R.G., Lopes N.B., Vicentini V.E.P. (2014). Antimutagenic and radioprotective activities of beta-carotene against the biological effects of iodine-131 radiopharmaceutical in Wistar rats. Genet. Mol. Res. GMR.

[33] Vrinceanu D., Dumitru M., Marinescu A., Serboiu C., Musat G., Radulescu M., Popa-Cherecheanu M., Ciornei C., Manole F. (2024). Management of Giant Thyroid Tumors in Patients with Multiple Comorbidities in a Tertiary Head and Neck Surgery Center. Biomedicines.

